# Energy transfer in complexes of water-soluble quantum dots and chlorin e6 molecules in different environments

**DOI:** 10.3762/bjnano.4.101

**Published:** 2013-12-13

**Authors:** Irina V Martynenko, Anna O Orlova, Vladimir G Maslov, Alexander V Baranov, Anatoly V Fedorov, Mikhail Artemyev

**Affiliations:** 1 Saint Petersburg National Research University of Information Technologies, Mechanics and Optics, Saint Petersburg, Russia; 2 Institute for Physico-Chemical Problems, Belarussian State University, Minsk, Belarus

**Keywords:** chlorin e6, FRET, quantum dots, track membrane

## Abstract

The photoexcitation energy transfer is found and investigated in complexes of CdSe/ZnS cationic quantum dots and chlorin e6 molecules formed by covalent bonding and electrostatic interaction in aqueous solution and in porous track membranes. The quantum dots and chlorin e6 molecules form stable complexes that exhibit Förster resonance energy transfer (FRET) from quantum dots to chlorin e6 regardless of complex formation conditions. Competitive channels of photoexcitation energy dissipation in the complexes, which hamper the FRET process, were found and discussed.

## Introduction

During the last decade, photophysical properties of the complexes formed by colloidal quantum dots (QDs) and organic molecules, in particular, complexes of QDs and tetrapyrrole compounds, were widely investigated [1–4]. The interest in complexes based on tetrapyrrole compounds has been sparked by their ability to generate singlet oxygen [5]. The singlet oxygen is used in different applications such as photodynamic therapy, blood plasma sterilization and wastewater treatment.

In QD/tetrapyrrole complexes the efficiency of singlet oxygen generation may be significantly increased, as compared with the free tetrapyrroles, due to an efficient photoexcitation energy transfer from the QD to the molecule. QDs have unique optical properties such as a broad absorption spectrum with extremely high extinction coefficient and high quantum yield of photoluminescence with the wavelength controlled by the QD size. It is very attractive to use QDs as an energy donor in complexes with organic molecules since the conditions for an effective FRET can be quite easily satisfied.

For effective functioning of these complexes as singlet oxygen generators, two conditions should be simultaneously fulfilled: 1) the ability of the tetrapyrrole molecules to generate singlet oxygen upon complex formation should be maintained and 2) the effective intracomplex photoexcitation energy transfer should occur.

In QD–tetrapyrrole complexes, a formation of competitive channels of nonradiative photoexcitation energy dissipation different from FRET may take place for both donor and acceptor [4]. The origin of these energy transfer channels is not completely understood. Several physical mechanisms have been proposed, for example, the photoinduced reversible electron transfer between QD and molecule, and the formation of QD photoluminescence deactivation centers at the place where the molecule is attached to the QD.

Chlorin e6 (Ce6) is one of the tetrapyrrole compounds widely used as a photosensitizer. Photophysical properties of complexes between QDs and chlorin e6 were discussed in [6–7]. For example, FRET in covalently linked QD-Ce6 conjugates in aqueous solution was demonstrated [6]. A strong quenching of the photoluminescence (PL) of Ce6 was also observed together with significant changes of the PL and absorption spectra of complexed Ce6 as compared with those of free Ce6. Similar quenching of the PL of Ce6 with increasing the molar concentration ratio of Ce6 and QDs (*n*) was also observed in [7]. At the same time, a conservation of photophysical properties of the tetrapyrrole component is extremely important, since the decrease in PL quantum yield (QY) of the tetrapyrroles is usually accompanied by a decrease in efficiency of singlet oxygen generation [4].

In this study we investigate the photophysical properties of QD–Ce6 complexes under variable conditions of formation such as the molar concentration ratio *n*, the binding type, the ambient environment and the size of the QDs in order to understand the intracomplex photoexcitation energy transfer processes like FRET and other competitive energy transfer mechanisms. Complexes were obtained in two different environments: in aqueous solution and in poly(ethylene terephthalate) track membranes that can be utilized as an element of microfluidic devices [8].

## Experimental

### Chemicals

Bis-*N*-methyl-D-glucamine salt of chlorin e6 (photosensitizer “Photoditazin”) was purchased from VETA Grand Ltd. Photoditazin has a QY of 9% in aqueous solution. Trioctylphosphine oxide (TOPO), cysteamine, 2-(dimethylamino)ethanethiol (DMAET), 1-ethyl-3-(3-dimethylaminopropyl)carbodiimide hydrochloride (EDAC) were purchased from Aldrich. Poly(ethylene terephthalate) (PET) membranes were obtained from FLNR JINR (Dubna, Russia).

### Quantum dot synthesis

All semiconductor quantum dots CdSe/ZnS with different core sizes (2.5 nm, 3.5 nm, and 5 nm) were synthesized using similar methods as previously described [9]. All QD samples have a QY of about 20% in hydrophobic solvents and 5–8% in aqueous solutions.

### Complex formation in aqueous solutions

To form water-soluble complexes of quantum dots and Ce6 molecules two methods of QD solubilization were used. In the case of covalent binding the hydrophobic CdSe/ZnS/TOPO QDs with a core diameter of 3.5 nm were initially solubilized by L-cysteine. In the second step, the L-cysteine molecules were replaced with molecules of hydroxy-terminated polyethylene glycol (PEG-OH)thiol and amino-terminated polyethylene glycol (PEG-NH_2_)thiol with a ratio of 3 to 1. This enables to obtain stable colloidal solutions of quantum dots. Then the covalent binding of the QD surface amino groups with the Ce6 carboxyl functional groups using EDAC as a cross-linking reagent was performed. Using PEG as an additional QD shell resulted in an increase of the average distance between the QD and Ce6 molecules in the complexes to ≈5.5 nm. For the complexes, formed via electrostatic interaction, the hydrophobic CdSe/ZnS/TOPO QDs with a core diameter of 5.0 nm were solubilized with DMAET molecules to provide a positive charge on the QD surface.

### Complex formation in the track membranes

The characteristics of PET track membranes are shown in Table 1. An ion-track technique is utilized for fabrication track pore membranes from thin polymer films [10]. Because of the etching, carboxyl groups are produced on the interior surface and in the loosened layer nearby the pore wall. The dissociation of the carboxyl groups in aqueous solutions leads to the appearance of negative charges on the track pore surface [11]. This gives the opportunity to passivate the inner surface of the pores with species that can react with carboxyl groups.

**Table 1:** PET track membranes characteristics.

Pore diameter, *d*	0.5 μm
Thickness, *l*	12 μm
Pore density, *p*	2.9·10^7^ cm^−2^
Pore direction (relative to the foil surface), 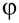	90°

We proposed to utilize these carboxyl groups for a step-by-step formation of the water soluble QD/Ce6 complexes in the membranes. At the first step, positively charged CdSe/ZnS/DMAET QDs with a core size of 2.5 nm were embedded into the membranes due to electrostatic interaction with the carboxyl groups on the inner surface and in the loosened layer on the track pore wall. Membranes with embedded QDs were impregnated by aqueous solutions of Ce6 for formation of the QDs/Ce6 complexes. In order to study the dependence of the optical properties of the complex components on the Ce6 concentration, the samples were immersed sequentially 10 times into the Ce6 solution for 5 minutes. After each immersion the membranes were removed from the Ce6 solutions, rinsed thoroughly with water and dried, then the static and time-resolved optical measurements were performed.

Since Ce6 molecules have carboxyl groups, these molecules are negatively charged in aqueous solution. Our experiments have shown that the embedding of Ce6 molecules into membranes without QDs does not occur due to the electrostatic repulsion of the carboxyl groups of the membrane and those of the Ce6. We replaced the carboxyl groups on the pore walls by amino groups with EDAC similar as described in [12]. This made it possible to create membranes with positive charges on the pore walls and to embed Ce6 molecules into membranes.

### Estimations of the FRET efficiency

In the Förster formalism, a distance dependence of the efficiency of FRET between the donor–acceptor (D–A) pair, *Q*_FRET_, is given with [13]:

[1]
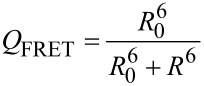


where *R*_0_ (Förster radius) is the D–A distance at which the transfer efficiency is 50%, and *R* is the D–A distance.

*R*_0_ can be calculated using the following equation:

[2]
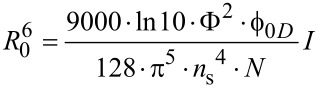


In this equation, Φ^2^ is the orientation factor, Φ_0_*_D_* is the quantum yield of the donor in the absence of quencher; *N* is the Avogadro number and *n*_s_ is the refractive index of the solvent. *I* is the overlap integral between the donor emission band and the acceptor absorption band:

[3]
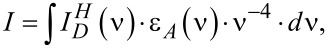


where 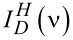 is the normalized PL spectrum of the energy donor; ε*_A_*(ν) is the absorption spectrum of the acceptor; ν is the wavenumber. Equation 1 does not take into account a possible appearance of additional nonradiative dissipation channels due to the complex formation. That is why we can use it only for estimation of the upper limit of the FRET efficiency for the donor–acceptor pair in the complexes. The energy transfer efficiency from donor to acceptor, 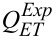, can be correctly estimated from the experimental data on the sensitized acceptor emission intensity [14–15], or the PL QY by using the following equation:

[4]
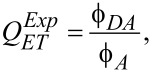


where Φ*_DA_* and Φ*_A_* are the PL QY of the acceptor sensitized with QDs and directly excited acceptor emission, respectively. There can be some difficulties in the direct measurement of the Φ*_DA_* value, because the contribution of directly excited molecule emission should be correctly accounted for. It also should be noted that the use of Equation 4 is only possible in cases when the molecule does not change its photophysical properties upon binding to a QD. Otherwise, Equation 4 should be modified and the energy transfer efficiency can be estimated from experimental data by using a formula similar to that reported in [16]:

[5]
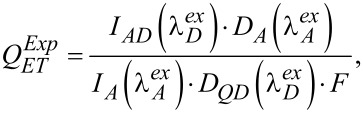


where *I_AD_* is the intensity of sensitized acceptor PL, *I_A_* is the intensity of acceptor PL directly excited by light; *D_A_* and *D_QD_* are the optical densities of the acceptor and donor at the excitation wavelengths of the PL. 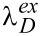 and 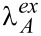 are the wavelengths of the exciting light. The values of 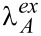 and 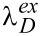 are usually chosen in such a way that a selective excitation of PL either of acceptor or of donor is performed, respectively. *F* is the efficiency of donor PL quenching:

[6]
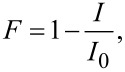


where *I* and *I*_0_ are the donor PL intensities in presence and in absence of the energy acceptor, respectively.

In the general case, when the complex formation results in appearance of additional nonradiative energy relaxation channels in the donor (QDs) or in a change of the acceptor PL quantum yield as compared to the free acceptor Equation 4 and Equation 5 are more correct for estimation of the energy transfer efficiency than Equation 1. For these equations mechanism of energy transfer from D–A (resulting in donor emission quenching and acceptor emission enhancing) is not strictly important. However, in the case when FRET is the only possible mechanism of energy transfer resulting in acceptor emission enhancing, the utilization of Equation 4 and Equation 5 provides a sufficient estimation of the FRET efficiency.

### UV–vis absorption and photoluminescence detection

A spectrophotometer, UV-Probe 3600 (Shimadzu) and a spectrofluorometer, Cary Eclipse (Varian) were used for steady-state absorption and PL measurements, respectively. A 475 nm (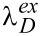) light was used for indirect excitation of the Ce6 PL. At this wavelength there is a local minimum of the Ce6 absorption while the QDs can be efficiently excited by the light. For direct excitation of the Ce6 PL, the light with a wavelength in the spectral range of 640–660 nm (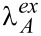), where a strong Q(I) band of Ce6 is located and the QD absorption is relatively small, was used. Time resolved luminescence measurements were performed with a laser scanning luminescent microscope, Micro Time 100 (Pico Quant), that allows registration of the luminescence decay in the 430–850 nm spectral range with 100 ps time resolution. A 80 ps pulse diode laser operated at 405 nm was used for the PL excitation.

## Results and Discussion

**Figure 1:**
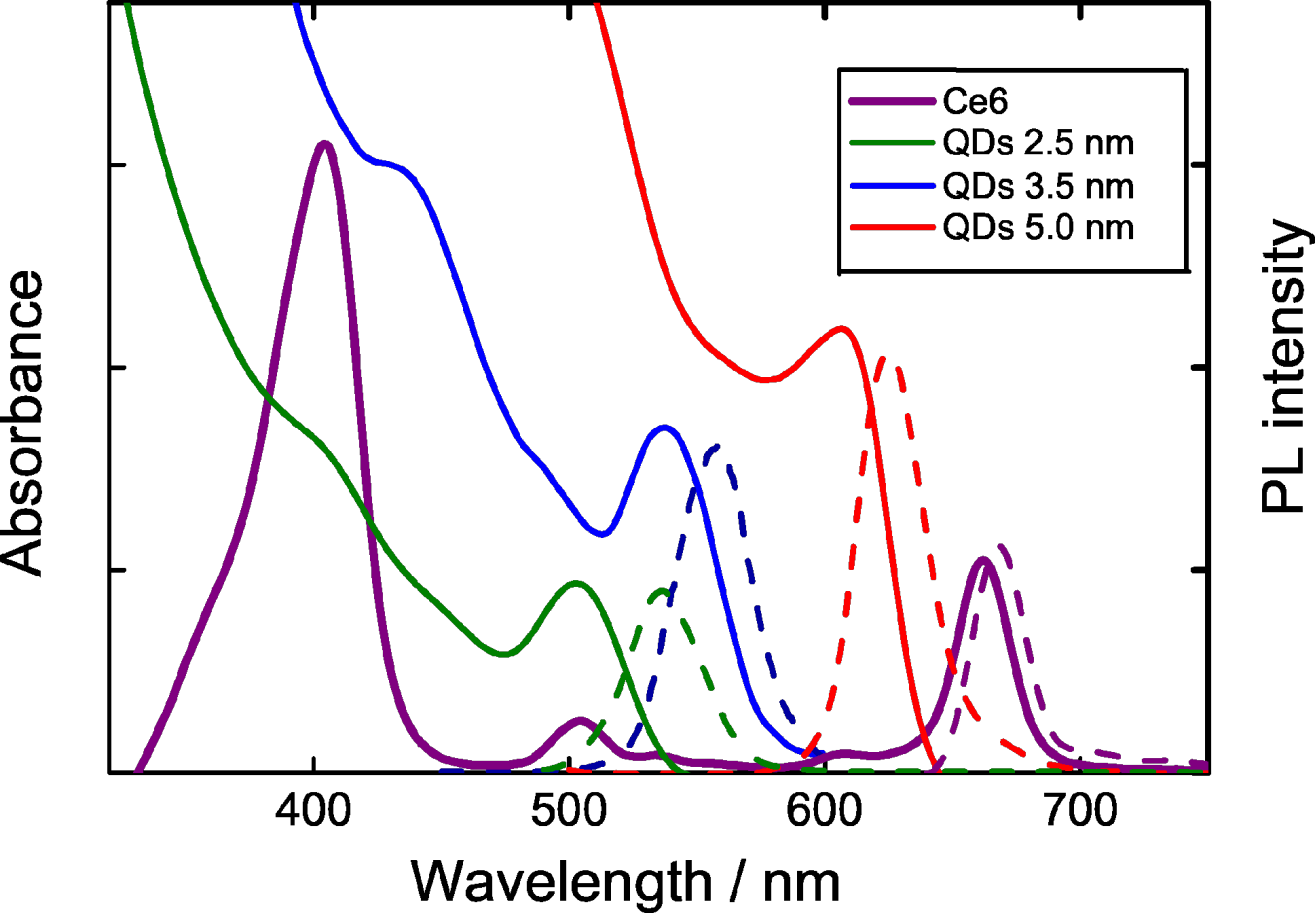
The absorption (solid line) and photoluminescence (dash line) spectra of QDs of different sizes and Ce6. The QD sizes are shown in the legend.

Figure 1 shows the PL and absorption spectra of QDs with core diameters of 2.5 nm, 3.5 nm, and 5.0 nm used in this study as well as the PL and absorption spectra of Ce6. In all cases the spectral overlapping of donor (QDs) PL with acceptor (Ce6) absorption needed for the FRET is satisfied.

The quenching of QD PL and sensitization of the Ce6 PL were observed in all prepared QDs–Ce6 complexes. These facts, which will be discussed in details below, indicate the presence of intracomplex energy transfer from QDs to molecules. An expected energy transfer efficiency can be easily estimated for the FRET process by using Equation 1 and Equation 2. Table 2 shows the calculated Förster radius (*R*_0_) and FRET efficiency (*Q*_FRET_) for various QD–Ce6 pairs using Equation 1 and Equation 2 with Φ_0_*_D_* = 1, *n_s_* = 1, and Φ^2^ = 2/3 [16]. We suppose that the distance between donor and acceptor is approximately equal to the dot radius, taking into account the size of the QDs, the thickness of the ZnS shell and the length of the solubilizer molecules.

**Table 2:** Förster distances, *R*_0_ and FRET efficiency, *Q*_FRET_ for QD–Ce6 complexes with different QD sizes.

Type of bonding	Covalent bonding	Electrostatic interaction in aqueous solution	Electrostatic interaction in PET membrane

QD size *d*, nm	3.5	5.0	2.5
Distance between donor and acceptor *R*, nm	5.5	2.0	1.75
Förster radius *R*_0_, nm	4.6	5.1	4.7
FRET efficiency *Q*_FRET_, %	27	98	98

### Covalently bonded QDs–Ce6 complexes

**Figure 2:**
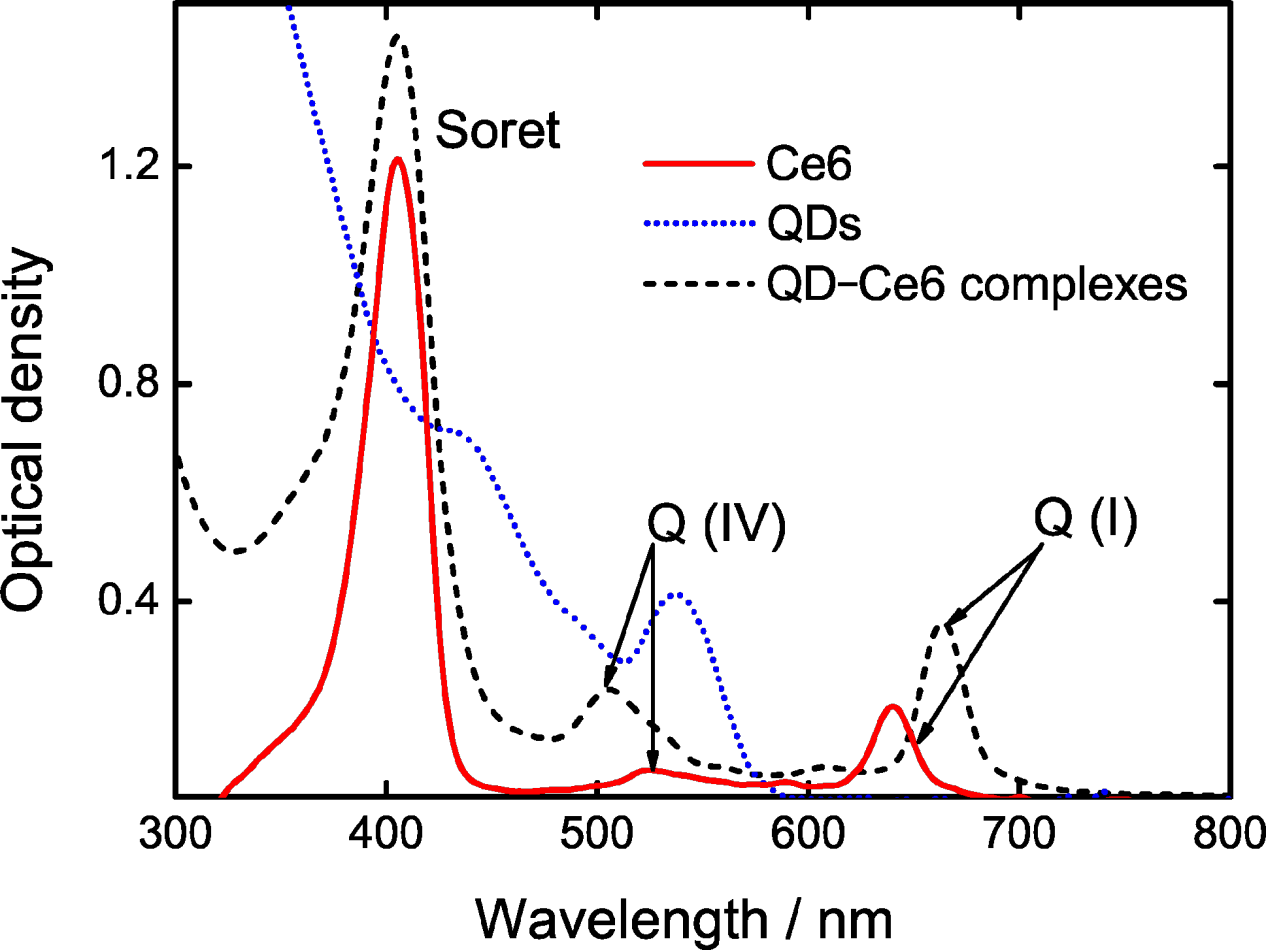
The absorption spectra of Ce6 (solid line), QDs (dotted line) and QD/Ce6 complexes (dashed line) in aqueous solution. The concentration of QDs: *C_QD_* ≈ 4 × 10^−7^ mol/L, *n* = *C_Ce_*_6_: *C_QD_* = 20. Characteristic absorption bands (Soret, Q(IV), and Q(I)) of the Ce6 are marked.

The absorption spectrum of QDs–Ce6 complexes, where the Ce6 is covalently bonded to quantum dots, and the spectra of the individual components of the complexes in aqueous solutions are shown in Figure 2.

The absorption spectrum of QDs–Ce6 complexes is not a simple superposition of pure QD and Ce6 absorption spectra at corresponding concentrations. Figure 2 shows that complex formation leads to changes in the absorption spectrum of Ce6 bound to QDs. Most pronounced changes were observed in the region of the Ce6 Q(I) band, which demonstrates a bathochromic shift to 662 nm. Also a hypsochromic shift of the Q(IV) of ≈20 nm, and a change in the bandwidth of the Soret band at ~400 nm were observed. Close changes in the Ce6 absorption were already reported for InP/ZnS/Ce6 complexes in dimethylformamide solution [6] and for Ce6 embedded into polyvinylpyrrolidone polymer chains [17]. It was concluded [17–18] that the observed modifications are a typical response to the changes of the Ce6 environment.

**Figure 3:**
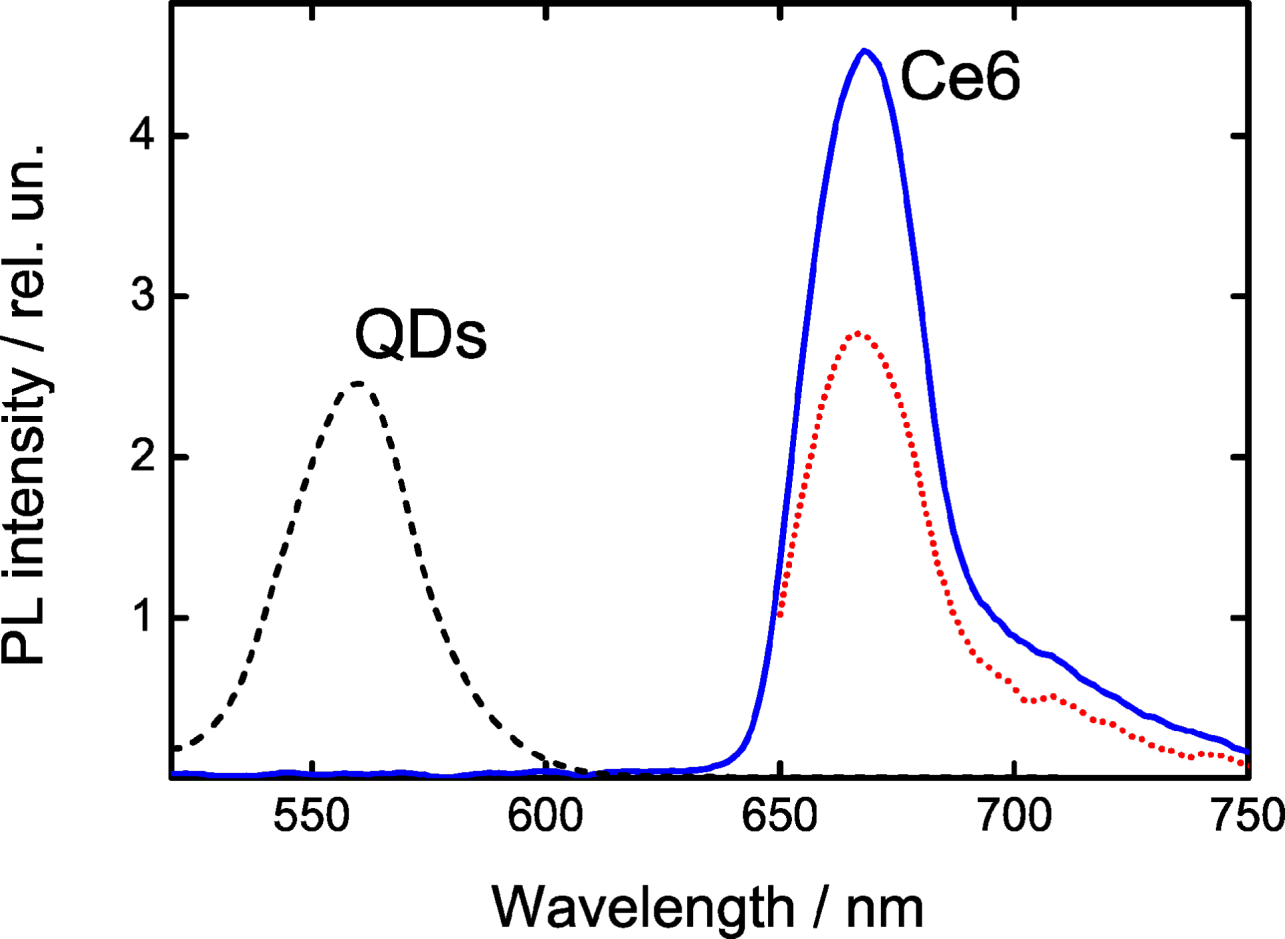
The PL spectra of covalent QDs–Ce6 complexes excited at 475 nm (solid line) and 640 nm (dotted line). The Ce6 PL intensity is normalized to optical density at excitation wavelength. The PL spectrum of QDs excited at 475 nm (dashed line) is shown for comparison.

To evaluate the efficiency of the energy transfer in the QD–Ce6 complexes using Equation 5, the PL of complexes was excited at two different wavelengths in order to selectively excite PL of either Ce6 or QDs. A 475 nm radiation was used for selective excitation of the QD PL within the QD–Ce6 complex since at this wavelength absorption of QDs was dominant as compared with that of Ce6. On the other hand, 640 nm light was used for selective Ce6 PL excitation to measure the Ce6 PL QY within the complex. For clarification of sensitized PL of Ce6 in these complexes, we normalized the experimental raw spectra to the optical density at the excitation wavelengths, i.e. 475 nm and 640 nm (see Figure 2, dashed line). The PL spectra presented in Figure 3 show that the complex formation results in complete QD PL quenching. At the same time, the Ce6 PL intensity, normalized to the Ce6 optical density at excitation wavelength, is ≈1.5 times higher for 475 nm excitation than for the 640 nm one that indicates a presence of sensibilized Ce6 luminescence. These facts show intracomplex FRET from QD to Ce6.

For this system, the energy transfer efficiency 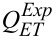 of 8%, calculated from experimental data with Equation 5, is significantly lower than its expected value *Q*_FRET_ of 27% estimated using the Förster formula (Equation 1). The relatively low *Q*_FRET_ value is caused by the large distance between the QD and Ce6 (≈5.5 nm) due to the use of modified polyethylene as a QD solubilizer.

Since the FRET efficiency reaches only 30% of its maximum value, we can assume that quenching of QD PL cannot be explained only by FRET, which is responsible only for 30% of the quenching. Therefore, a presence of competitive channels of a nonradiative photoexcitation energy dissipation in QDs should be taken into account. On the other hand, QY of Ce6 PL in the complexes is about 3 times lower than that in Ce6 bound with PVP [17] and 2 times lower than that for Ce6 in aqueous solutions. Appearance of new channels of Ce6 energy dissipation due to perturbation of the molecule under complexing or aggregation of chlorin e6 [17] on the surface of QDs may be responsible for the QY reduction.

### QDs–Ce6 complexing due to electrostatic interaction in aqueous solution

It is known that the FRET efficiency in the QD–molecule complexes may depend on the number of acceptor molecules on the surface of QD [14]. This effect can be studied in case of QD–Ce6 complex formation by electrostatic interaction of oppositely charged solubilizer molecules on the QD surface and carboxyl groups of Ce6. These complexes can be easily formed by simple mixing solutions of the components. Despite the instability of these complexes and their tendency to dissociation, they are supposed to be good model objects to explore the dependencies of FRET efficiency and Ce6 QY on *n* in the complexes by spectral luminescence methods.

**Figure 4:**
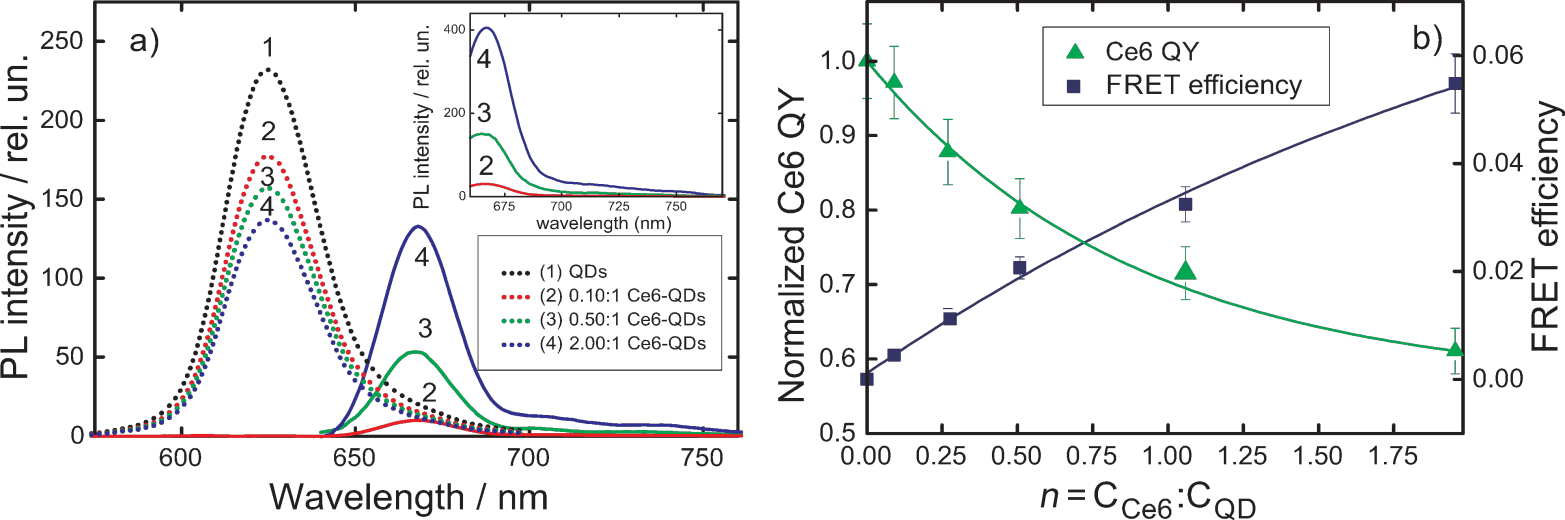
Optical properties of QD–Ce6 complexes formed by electrostatic interactions in aqueous solution as a function of *n*. (a) Evolution of PL spectra of 5 nm QDs (dotted line) and sensibilized luminescence of Ce6 (solid line) extracted from PL spectra of the QD–Ce6 complexes. The PL excitation wavelength is 475 nm. The intensity of the Ce6 emission is multiplied by 2. The inset shows PL spectra of the QD–Ce6 complexes with excitation at 640 nm. (b) FRET efficiency, estimated using Equation 5, and Ce6 QY as a function of *n*.

The samples for studies were prepared by stepwise increasing the Ce6 concentration in solution of QD and Ce6, keeping the concentration of QDs constant (*C_QD_* ≈ 5 × 10^−7^ mol/L). Band positions in absorption and PL spectra of Ce6 in these complexes coincided with those in covalently bound QDs–Ce6 complexes and did not depend on the Ce6 concentration. An increase of the relative concentration of Ce6 led to quenching QD PL and to appearance of the sensibilized luminescence of Ce6. This is illustrated in Figure 4a where examples of PL spectra of QDs and Ce6 excited at 475 nm are presented. The inset in Figure 4a shows the *n*-dependence of intrinsic Ce6 PL excited at 640 nm. Analysis of these data demonstrates that the intensity of intrinsic Ce6 PL increases slower with increasing *n*, i.e., Ce6 PL QY decreases with growth of *n*, as it shown in Figure 4b. It was found that QD PL lifetimes remained the same upon increasing *n*. This indicates that the binding of QD to the Ce6 molecule leads to total QD PL quenching.

Figure 4b shows that although the FRET efficiency rapidly increases with *n*, its value 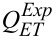 calculated using Equation 5 does not exceed 6%. It is significantly lower than the theoretical value of 98% (Table 2) and indicates the presence of energy dissipation channels different from FRET. Similar quenching of Ce6 PL with an increase of *n* was also observed in [7]. The increasing Ce6 concentration in complexes of CdSe/ZnS QD and Ce6 formed by electrostatic interaction led to strong Ce6 PL quenching that allows to suggest a formation of competitive channels of Ce6 energy dissipation.

Origin of the dependencies of the FRET efficiency and QY of Ce6 PL on *n* is not quite clear and requires an additional analysis. When molar ratio *n* varied from 1:0.1 to 1:1, the average number of Ce6 molecules per QD is less than one. It means that the number of complexes with more than one Ce6 molecules is negligible. This excludes an interaction between close Ce6 molecules located on the QD surface as a reason for decreasing of Ce6 QY. As a first approach, we assume that this dependence might be caused by QD aggregation in aqueous solution. To check this assumption QD–Ce6 complexes formed by electrostatic interaction in polymer track membranes were studied.

### QDs–Ce6 complexes in track membranes

**Figure 5:**
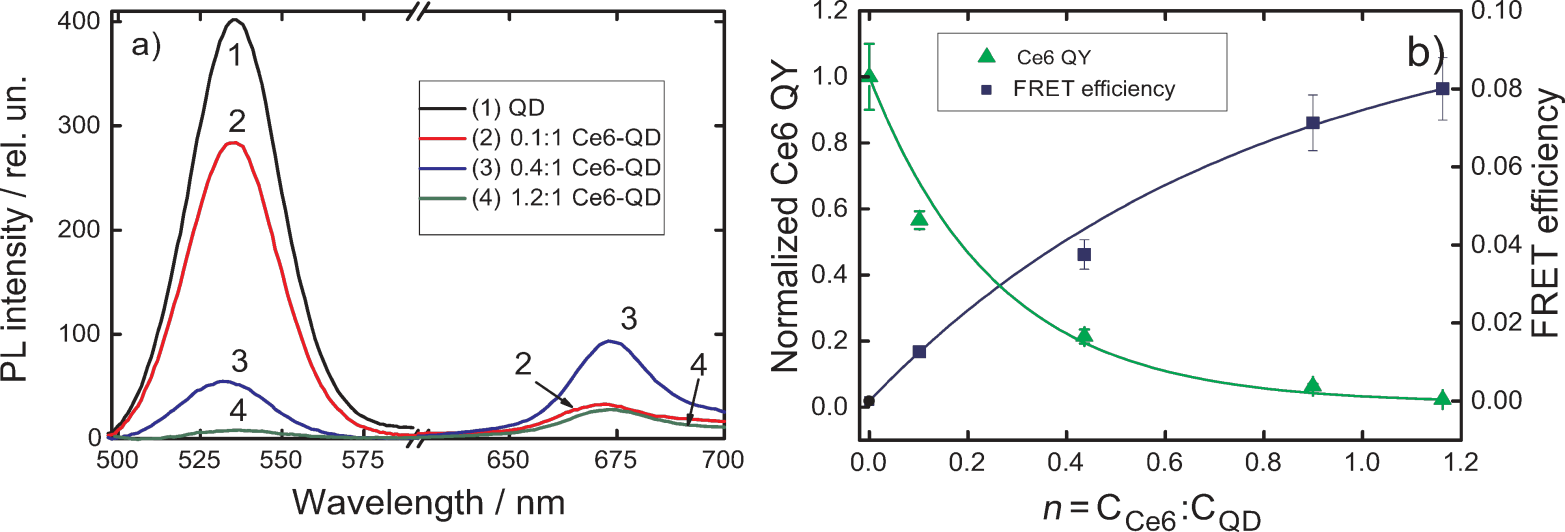
Optical properties of QD–Ce6 complexes formed by electrostatic interaction in the PET membrane as a function of *n*. (a) Evolution of PL spectra of 2.5 nm QDs and Ce6 complexes. PL excitation wavelength is 475 nm. (b) FRET efficiency, estimated using Equation 5 and Ce6 QY as a function of *n*.

Aggregation of the quantum dots in solution is one of the possible reason of nonradiative dissipation of the QD excited state [19]. Embedding of cationic quantum dots to a pore wall layer via carboxyl groups of the membrane can prevent spontaneous aggregation of QDs [20]. The described embedding of Ce6 allows the creation of the QD–Ce6 complexes in the track membrane via electrostatic interaction. We found that for such complexes an increase of *n* leads to a complete quenching of QD PL and to a fatal decrease of the PL intensity of Ce6 molecules bound to QDs (Figure 5a). The QD–Ce6 complexes in the track membranes demonstrate the same dependencies of FRET efficiency and QY of Ce6 on *n* as in aqueous solution, i.e., when *n* increases the FRET efficiency increases while Ce6 QY decreases (Figure 5b). It should be noted that for *n* = 1.2 the Ce6 QY reduced nearly to zero.

Summarizing obtained experimental data, we can assume that origin of dependence of FRET efficiency and QY of Ce6 on *n* in QD–Ce6 complexes is irrelevant with aggregation of QDs and Ce6.

The experimental data demonstrate that nonradiative photoexcitation energy dissipation occurs in all types of QD–Ce6 complexes and their contribution to the intracomplex energy transfer varies from 70% in covalently bonded complexes to ≈90% in complexes formed by electrostatic interactions and may be responsible for QD and Ce6 PL quenching.

Electron transfer is the other possible mechanism of the QD PL quenching in the QD–organic molecule complexes. This mechanism is efficient if either both LUMO and HOMO or one of these orbitals of the quencher molecule is located within the energy gap of the CdSe QD. In this case the photoexcited electron or hole tunnels from the QD core through the shell and localizes at the LUMO or HOMO of the quenching molecule, respectively. These mechanisms of the tetrapyrrole PL quenching in complexes with QDs have been proposed [21].

**Figure 6:**
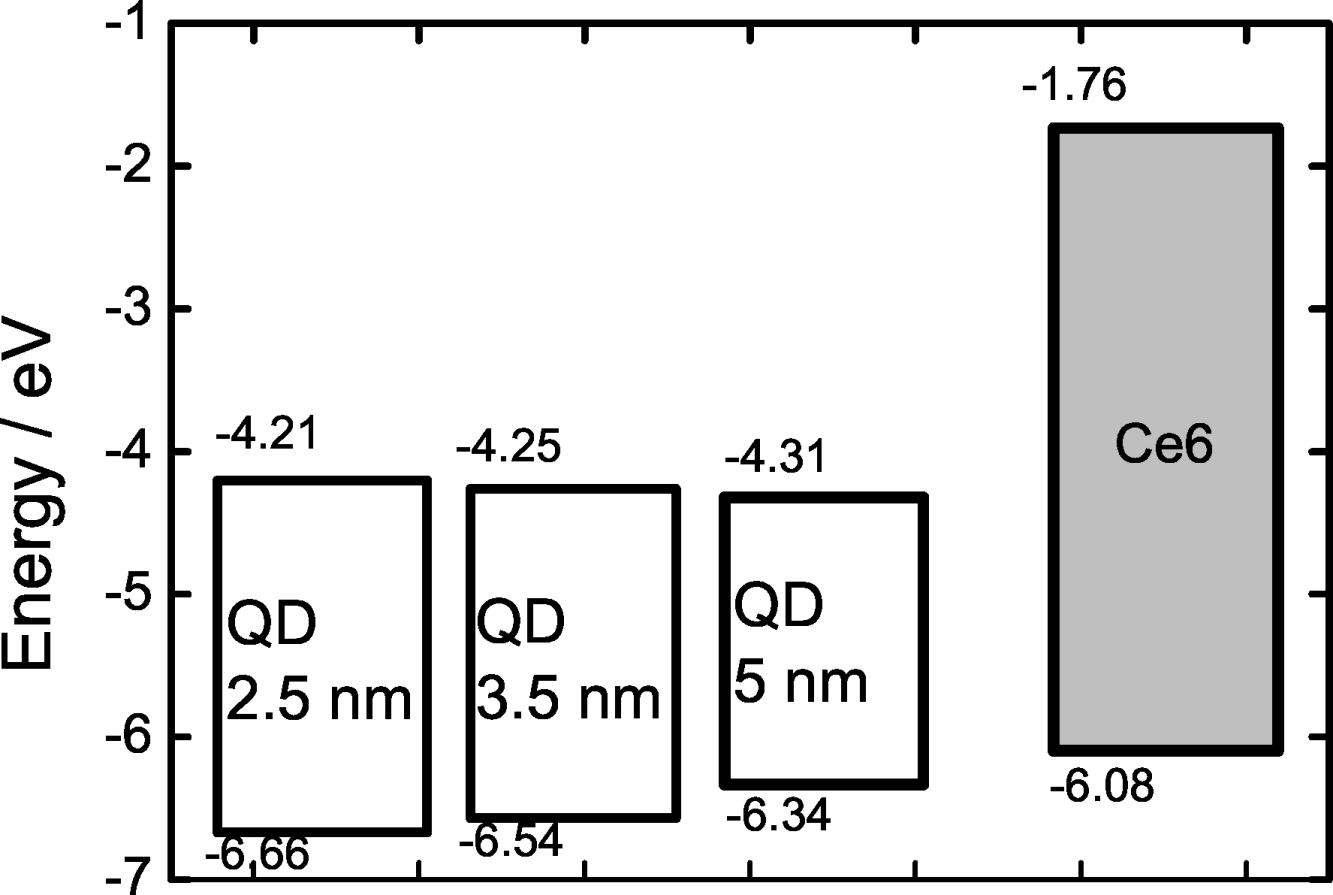
Energy level diagram of CdSe/ZnS QDs used in this work and Ce6 from [22]. The size of QD is shown in corresponding boxes.

The analysis of relative positions of the energy levels of QDs and chlorin e6, presented in Figure 6, shows that in complexes with CdSe/ZnS quantum dots a photoinduced electron transfer from Ce6 to the QDs conduction band is possible.

The electron transfer is a more short-range process than FRET because its efficiency drops exponentially with the D–A distance. In the case of the studied covalently linked complexes the D–A distance is too large for effective electron transfer. In agreement with this, FRET contribution to the intracomplex energy transfer in this type of complexes is larger than in complexes with electrostatic interaction (30% and 5% respectively). At the same time, a relatively small FRET contribution in covalently linked complexes, where electron transfer is excluded, indicates the existence of additional channels in QD–Ce6 complexes different from the electron transfer.

## Conclusion

The photophysical properties of complexes of CdSe/ZnS cationic quantum dots and chlorin e6 molecules formed by covalent bonding and electrostatic interaction in aqueous solution and PET membranes were investigated. It was found that interactions between quantum dots and chlorin e6 molecules in the QD–Ce6 systems lead to quenching of the quantum dots PL and to sensitizing the chlorin e6 PL. FRET from QDs to Ce6 was observed. Values of the FRET efficiencies estimated from the experimental data for all types of the complexes were low as compared with theoretically predicted for these D–A pairs. This fact and the essential decrease of the Ce6 photoluminescence QY by complex formation indicate that additional channels of nonradiative energy dissipation in QDs and/or in Ce6 should be taken into account. The photoinduced electron transfer from Ce6 to the QDs conduction band was considered as possible mechanism of nonradiative photoexcitation energy dissipation. However, the data analysis also indicated that additional mechanisms of nonraditive energy dissipation, like, e.g., energy transfer from Ce6 to local energy states inside the CdSe bandgap [23], have to be considered to better match our experimental results.
